# Reference Values of Handgrip and Lower Extremity Strength for Vietnamese Men and Women: The Vietnam Osteoporosis Study

**DOI:** 10.1002/jcsm.13689

**Published:** 2025-01-10

**Authors:** Kiet T. Do, Duy K. Hoang, Quan N. Luong, Huy G. Nguyen, An T. Do, Lan T. Ho‐Pham, Tuan V. Nguyen

**Affiliations:** ^1^ Department of Internal Medicine Le Van Thinh Hospital Ho Chi Minh City Vietnam; ^2^ Saigon Precision Medicine Research Center Ho Chi Minh City Vietnam; ^3^ School of Biomedical Engineering University of Technology Sydney Sydney Australia; ^4^ Department of Internal Medicine Vinmec Central Park International Hospital Ho Chi Minh City Vietnam; ^5^ BioMedicine Research Center Pham Ngoc Thach University of Medicine Vietnam; ^6^ Tam Anh Research Institute Ho Chi Minh City Vietnam; ^7^ School of Population Health UNSW Medicine UNSW Sydney Sydney Australia

**Keywords:** handgrip strength, lower extremity strength, muscle strength, reference values, Vietnam Osteoporosis Study

## Abstract

**Background:**

Falls and sarcopenia are significant public health issues in Vietnam. Despite muscle strength being a critical predictor for these conditions, reference data on muscle strength within the Vietnamese population are lacking.

**Purpose:**

To establish the reference ranges for muscle strength among Vietnamese individuals.

**Methods:**

The study involved 4096 individuals, including 1419 men and 2677 women aged 18 years and above, from the Vietnam Osteoporosis Study. Muscle strength was assessed using a Baseline hand dynamometer for handgrip strength and a Back‐Leg‐Chest dynamometer for leg strength. We calculated mean values, standard deviations, interquartile ranges, and peak muscle strength (*p*MS) for both handgrip and leg strength across various ages. Reference curves were created with the Generalised Additive Model for Location Scale and Shape, and polynomial regression models were employed to analyse the relationship between muscle strength and age.

**Results:**

Advancing age was significantly associated with lower muscle strength. Peak muscle strength typically occurred between ages 30 and 40, with earlier peaks in women, especially in leg strength. Men consistently showed higher muscle strength than women, with variations depending on the measurement site. Specifically, average handgrip strength was 36.4 kg ± 8.4 (mean ± SD) for men and 23.2 kg ± 6.0 for women (*p* < 0.001). Leg strength averaged 63.9 kg ± 27.2 for men and 29.5 kg ± 13.9 for women (*p* < 0.001). Additionally, we produced a percentile chart illustrating muscle weakness ranges based on the 25th percentile of muscle strength and the appendicular skeletal muscle mass index (ASMI) for the Vietnamese population.

**Conclusion:**

These data provide reference ranges for evaluating muscle strength in the Vietnamese population, offering crucial insights for identifying individuals at risk of falls or sarcopenia in clinical settings.

## 1 Introduction

Muscle strength is crucial for overall health and the performance of everyday activities. When muscle strength decreases, marked by muscle weakness, it not only predicts falls but also correlates with higher rates of hospitalisation [1], depression [2], cognitive impairment [3] and increased mortality [4]. These issues are integral to the diagnosis of sarcopenia, a condition defined by reduced muscle mass and function [5, 6]. The impact of sarcopenia and related falls is a major public health issue in both developed and developing nations. For instance, in Vietnam, the prevalence of sarcopenia stands at 14% [7], closely mirroring the 15% observed in the United States [8]. Globally, sarcopenia affects between 10% and 27% of populations, depending on the diagnostic criteria and thresholds applied [9]. Annually, around 646 000 deaths worldwide are attributed to falls, ranking them as the second leading cause of accidental death. Additionally, falls significantly shorten life expectancy and resulted in over $50 billion in costs in 2015 alone [10].

Both the European Working Group on Sarcopenia in Older People (EWGSOP2) [11] and the Asian Working Group for Sarcopenia (AWGS) 2019 [12] highlight the importance of muscle strength and muscle mass in sarcopenia diagnosis. However, a critical challenge in clinical practice is the need for robust, ethnicity‐specific normative reference values for muscle strength in low‐ and middle‐income countries (LMICs) [13, 14]. Currently used cut‐off points for muscle strength are often derived from studies conducted in high‐income countries, potentially leading to misdiagnosis in the LMIC population [15].

In Vietnam, the ageing population trend, alongside lifestyle shifts and increasing life expectancy [16], contributes to a faster decline in muscle strength. This situation underscores the importance of focusing on muscle strength and addressing its related health issues. Despite the urgent need for precise measurement and management of muscle strength, there is a significant scarcity of documented reference values specific to the Vietnamese population. This study seeks to fill that gap by establishing normative reference values for muscle strength among Vietnamese individuals. Our comprehensive approach aims to establish ethnic‐specific thresholds for muscle strength and ASMI to facilitate early diagnosis of sarcopenia and enable targeted interventions.

## 2 Study Design and Methods

### 2.1 Study Design

This study was part of the Vietnam Osteoporosis Study (VOS) project, in which details and protocol have been published previously [17]. The study was designed as a population‐based investigation, encompassing participants from both Ho Chi Minh City and its surrounding districts. The study was approved by the research and ethics committee of People's Hospital 115 on 6 August 2015 under approval number 297/BV‐NCKH. Compliance with the ethical standards of the Declaration of Helsinki was ensured, as all participants gave written informed consent.

The inclusion criteria were broad, encompassing men and women aged 18 years and older who voluntarily joined the study. The exclusion criteria excluded individuals with cognitive impairments or cancer, those who declined to provide informed consent or those unable to perform the clinical tests due to physical limitations.

### 2.2 Anthropometric Measurements

Height and body weight were measured by an electronic portable, wall‐mounted digital scale (Seca Model 769; Seca Corp., CA, USA) without shoes, hats, ornaments or heavy layers of clothing. Body mass index (BMI) was calculated as weight (kg) divided by the square of height (kg/m^2^).

### 2.3 Body Composition Measurements

Lean mass and fat mass were measured by dual‐energy x‐ray absorptiometry (DXA) using a Hologic Horizon densitometer (Hologic Corp., Bedford, MA, USA) with a standard adult's whole‐body scan mode. The Hologic software Version 12.6 was used to analyse lean and fat mass (kg). Fat mass and lean mass were derived from the whole‐body scan. The skeletal muscle mass is divided into the trunk and appendicular musculature. The ASMI was calculated as the sum of appendicular skeletal muscle mass over height squared (unit kg/m^2^).

### 2.4 Handgrip Strength Measurement

We used a Baseline hand dynamometer (HiRes Gauge: ER 300 lb. Capacity, 3B Scientific, Atlanta, GA, USA) to measure the handgrip strength (HGS) of both the right and left hands in each participant. The results were quantified in kilograms, with the smallest measurable value set at 0.1 kg for handgrip. Before measurements, participants engaged in a 5‐min warm‐up to ensure accuracy in HGS. The dominant hand of each participant was identified, and the handles of the handgrip dynamometer were adjusted to fit their hand size optimally. Participants were then positioned upright with a flexed elbow at 90°. With the palm facing inward, individuals were instructed to exert maximum force on the dynamometer handle for 3 s without looking at the gauge or sensing the grip movement. Two measurements were taken for each hand, with a 1‐min rest interval, and the highest recorded value was used for analysis. Participants were instructed not to use their other hand during measurement, with a minimum 1‐min rest between measurements [18].

### 2.5 Leg Strength Measurement

The Baseline Back‐Leg‐Chest (BLC) dynamometer (Oversize Platform: 660 lb. Capacity, 3B Scientific) was used to assess leg strength. The results were measured in kilograms, with the smallest measurable value set at 0.5 kg for leg strength. Before the assessment, participants engaged in a 5‐min warm‐up. Participants positioned themselves on a base with feet wider than shoulder–width apart and knees bent to 120°–140°. While maintaining a straight back, braced hips and core, participants were instructed to pull themselves upward using their legs and sustain the position without leaning back for as long as possible. The chain length was adjusted to accommodate height differences or vary the point of force application. The test was performed twice, with a 1‐min rest interval between measurements. The highest recorded value was used for analysis. Individuals were instructed not to use their arms to assist in the test [19].

### 2.6 Definition of Low Muscle Strength and ASMI

In this study, we used the criteria outlined by the AWGS 2019 to classify sarcopenia based on the lowest quintile of lean mass and grip strength, which were derived from the present study. Specifically, sarcopenia was identified in men with ASMI < 7.0 kg/m^2^ and grip strength < 28 kg and in women with ASMI < 5.4 kg/m^2^ and grip strength < 18 kg [12]. In addition, sarcopenia was defined based on the Vietnamese reference values, where individuals falling below the lowest percentiles of ASMI and grip strength were confirmed to have sarcopenia. These percentiles were determined by fitting a function to the data.

### 2.7 Statistical Analysis

Data were summarised in terms of mean and standard deviation for continuous variables and proportion for categorical variables. An unpaired *t*‐test tested the difference in muscle strength parameters between men and women for continuous data or a chi‐squared test for categorical data.

To construct reference ranges for a continuous variable, we used the median (*m*) and standard deviation (*s*) of the variable using the simple formulation *m ± ks*, where *k* is the quantile value of a percentile. Polynomial regression models, extending up to the third degree, were employed to assess muscle strength in the dominant hand, non‐dominant hand and leg concerning age using the formula MS = α + β₁(age) + β₂(age)^2^ + β₃(age)^3^, where MS is a measure of muscle strength, α represents the intercept and β₁, β₂ and β₃ are regression parameters estimated from observed data.

As each body composition parameter depends on gender and age, we extended the formulation to account for each gender and age using the Generalised Additive Model for Location Scale and Shape (GAMLSS) method [20]. GAMLSS extends the lambda–mu–sigma method, where ‘lambda’ is the skewness, ‘mu’ is the median and ‘sigma’ represents the vector of parameters accounting for a variable. A GAMLSS model requires a fitting distribution, and in this analysis, we specified the Box–Cox–t as the link distribution, with the median being the location parameter. Age was considered a ‘predictor’ in this model, and because age is a continuous variable, we used a linear model for each parameter. Under the normal assumption, both μ and σ are linearly dependent on age. The R package ‘gamlss’ was used to estimate model parameters.

Mean, standard deviation values, interquartile range (from the 5th to the 95th percentiles) and peak muscle strength (*p*MS) for HGS, leg muscle strength and ASMI were computed within each 10‐year age interval, starting from 18 years of age. The reference percentiles showed the 5th–95th percentiles of handgrip, leg strength and ASMI across ages for both genders.

To estimate the prevalence of sarcopenia, we utilised Vietnamese reference data and the criteria outlined in the AWGS 2019. The entire analysis was conducted using R statistical software (Version 4.3.2), with the level of statistical significance set at 5% [21].

## 3 Results

The study included 4096 individuals (1419 men and 2677 women) aged 18 and over (Table 1). Among those aged 40 and above, men made up approximately 31% of the group. The majority, with 85.3% of men and 88.6% of women, reported their right hand as their dominant hand. Generally, men exhibited greater muscle strength than women, with men's HGS being about 37.5% higher than that of women, and their leg strength nearly twice as high. Accordingly, ASMI was roughly 33% higher in men compared to women. The data also showed that 43% of women and 50.6% of men were classified as overweight or obese.

**TABLE 1:** General characteristics of 2677 women and 1419 men participants.

Characteristics	All (*N* = 4096)	Women (*N* = 2677)	Men (*N* = 1419)	*p*
Age (years)	46.8 (14.6)	47.8 (14.3)	44.8 (15.1)	< 0.001
Age group (*N*)				< 0.001
18–29	671 (16.4%)	376 (14.0%)	295 (20.8%)	
30–39	563 (13.7%)	333 (12.4%)	230 (16.2%)	
40–49	916 (22.4%)	628 (23.5%)	288 (20.3%)	
50–59	1213 (29.6%)	827 (30.9%)	386 (27.2%)	
60–69	518 (12.6%)	358 (13.4%)	160 (11.3%)	
70–79	173 (4.22%)	128 (4.78%)	45 (3.17%)	
80+	42 (1.03%)	27 (1.01%)	15 (1.06%)	
Height (cm)	157 (7.76)	153 (5.44)	164 (6.05)	< 0.001
Weight (kg)	56.5 (9.99)	53.2 (8.2)	62.8(10.0)	< 0.001
BMI (kg/m^2^)	22.9 (3.33)	22.7 (3.33)	23.3 (3.3)	< 0.001
BMI group (*N*)				< 0.001
Underweight	92 (2.25%)	60 (2.25%)	32 (2.26%)	
Normal	2132 (52.1%)	1464 (54.8%)	668 (47.1%)	
Overweight	1499 (36.7%)	934 (35.0%)	565 (39.8%)	
Obese	367 (8.97%)	214 (8.01%)	153 (10.8%)	
Lean mass (kg)	35.5 (7.47)	31.1 (3.81)	43.8 (5.35)	< 0.001
ASMI (kg/m^2^)	6.12 (1.15)	5.50 (0.70)	7.30 (0.88)	< 0.001
Right hand (*N*)	3582 (87.5%)	2372 (88.6%)	1210 (85.3%)	< 0.001
Grip strength (kg)				
Dominant hand (kg)	27.8 (9.36)	23.2 (5.96)	36.4 (8.42)	< 0.001
Non‐dominant hand (kg)	26.2 (8.96)	21.7 (5.74)	34.5 (7.94)	< 0.001
Leg strength (kg)	41.8 (25.7)	29.5 (13.9)	63.9 (27.2)	< 0.001

*Note:* Values are mean (standard deviation). *p* value was derived from an unpaired *t*‐test and chi‐squared test for difference between men and women.

### 3.1 Association Between Muscle Strength, ASMI and Age

The relationship between muscle strength and age was best described by a third‐degree polynomial regression model, as indicated by the parameters in the Supporting Information. Based on this model, we estimated *p*MS for both the dominant and non‐dominant hand (DH and NDH), as well as for the leg and ASMI for both men and women across age groups (Table 2). For handgrip, the onset of the *p*MS interval was delayed in women (ages 40–49) compared to men (ages 30–39). However, for leg strength, the onset of this interval was delayed in men (ages 40–49) compared to women (ages 30–39). Notably, there was a consistent pattern of higher *p*MS in men compared to women, with the disparity varying by muscle site. Specifically, in the dominant hand, *p*MS in men (38.3 kg ± 8.5) was about 55% higher than in women (24.7 kg ± 5.8); a similar pattern was observed in the non‐dominant hand. Additionally, leg strength in men (69.3 kg ± 27.5) was approximately double that in women (32.3 kg ± 15.7). ASMI reached peak value between ages of 30 and 39 in men, whereas in women, the peak occurred later, between ages of 40 and 49. ASMI in men (7.5 kg/m^2^ ± 0.8) was roughly one‐third higher than in women (5.6 kg/m^2^ ± 0.7).

**TABLE 2:** Centiles and mean of grip strength, leg strength and appendicular skeletal muscle index of men stratified by age groups.

Parameter	Group	Centiles					Mean	SD
		5th	25th	50th	75th	95th		
Dominant handgrip strength (kg)	18–29 years	24	32	38	44	50	38.1	8.1
	30–39 years	24	32	38	44	50	38.3	8.5
	40–49 years	24	32	39	43	50	37.8	7.8
	50–59 years	21.4	30	36	42	50	35.9	8.4
	60–69 years	20	28	32	38	46	32.9	7.5
	70–79 years	18.1	24	30	32	37.7	28.3	6.4
	80+ years	17.1	24	29	32.5	35.8	28.1	6.7
Non‐dominant handgrip strength (kg)	18–29 years	22.8	30	36	41	48.2	35.8	7.7
	30–39 years	22	30	36	42	48	36.2	7.7
	40–49 years	23	31	38	42	48	36.2	7.6
	50–59 years	20	28.8	34	40	47.6	33.9	7.9
	60–69 years	20	26	32	36	42	31	7
	70–79 years	16.4	23.5	28	30.5	38	27.2	6.4
	80+ years	18.8	22.5	28	31	34.5	26.9	5.9
Leg strength (kg)	18–29 years	30	45	65	85	120	66.5	26.9
	30–39 years	30	50	65	82	110	67.3	26.5
	40–49 years	30	50	65	85	120	69.3	27.5
	50–59 years	24	40	60	80	118	62.5	27.3
	60–69 years	20	38	50	70	90	53.4	24.6
	70–79 years	20	31	50	60	91	48.8	23
	80+ years	20	30	31.5	58.8	73.5	41.9	19.4
Appendicular skeletal muscle index (kg/m^2^)	18–29 years	6	6.7	7.3	7.9	8.8	7.3	0.9
	30–39 years	6.3	6.9	7.4	8.1	8.8	7.5	0.8
	40–49 years	6.3	6.8	7.4	8	8.9	7.4	0.8
	50–59 years	5.9	6.8	7.3	7.8	8.7	7.3	0.9
	60–69 years	5.7	6.5	7	7.5	8.3	7	0.8
	70–79 years	5.4	6	6.5	7.3	8.1	6.6	0.9
	80+ years	4.9	6	6.1	6.5	7.4	6.2	0.8

### 3.2 Muscle Strength by Age

Tables 2 and 3 present normative values for HGS, leg strength and ASMI across age groups for both men and women. Mean values ± SD along with the 5th, 25th, 50th, 75th and 95th percentiles are reported. The percentile values for HGS and leg strength indicate three periods in life: an increase to peak in early adulthood (18–30 years), maintenance through midlife (30–40 years) and subsequent decline from midlife onwards (40+ years).

**TABLE 3:** Centiles and mean of grip strength, leg strength and appendicular skeletal muscle index of women stratified by age groups.

Parameter	Group	Centiles					Mean	SD
		5th	25th	50th	75th	95th		
Dominant handgrip strength (kg)	18–29 years	14.9	20	24	29	34	24.5	5.8
	30–39 years	14	20	24	28.2	34	24.4	6.0
	40 to 49 years	14	20.2	24	29	34	24.7	5.8
	50–59 years	12	19	22	26	32	22.6	5.9
	60–69 years	12	18	22	24	29.9	21.3	5.2
	70–79 years	10	16	20	23	28	19.3	5.4
	80+ years	12	14	15.5	20.5	24	16.9	4.3
Non‐dominant handgrip strength (kg)	18–29 years	14	19	22	26	32	22.6	5.5
	30–39 years	12	20	22	26	32	22.8	5.9
	40–49 years	14	20	23	28	32	23.3	5.7
	50–59 years	12	18	22	25	30	21.2	5.7
	60–69 years	10	18	20	23.8	28	20	4.9
	70–79 years	10	15.2	18.5	22	26.9	18.5	5.2
	80+ years	10	12	16	18	21.6	15.6	4
Leg strength (kg)	18–29 years	10	20	30	40	55	30.3	13.8
	30–39 years	10	20	30	40	60	32.3	15.7
	40–49 years	10	20	28	40	60	30.4	14.7
	50–59 years	10	20	29	38	52.6	29.1	13.2
	60–69 years	10	20	25	35	50	27	12.6
	70–79 years	10	15	22	30	43.4	25.1	11.4
	80+ years	13.5	20	20	24.5	26.5	21	4.6
Appendicular skeletal muscle index (kg/m^2^)	18–29 years	4.3	4.8	5.1	5.6	6.5	5.2	0.6
	30–39 years	4.6	5.1	5.4	5.8	6.6	5.5	0.6
	40–49 years	4.7	5.2	5.6	6	6.9	5.6	0.7
	50–59 years	4.5	5.1	5.5	5.9	6.8	5.5	0.7
	60–69 years	4.4	5	5.4	5.9	6.7	5.5	0.7
	70–79 years	4.4	5	5.4	5.9	6.6	5.5	0.7
	80+ years	4.1	4.7	5.1	5.8	6.5	5.2	0.8

**FIGURE 1:**
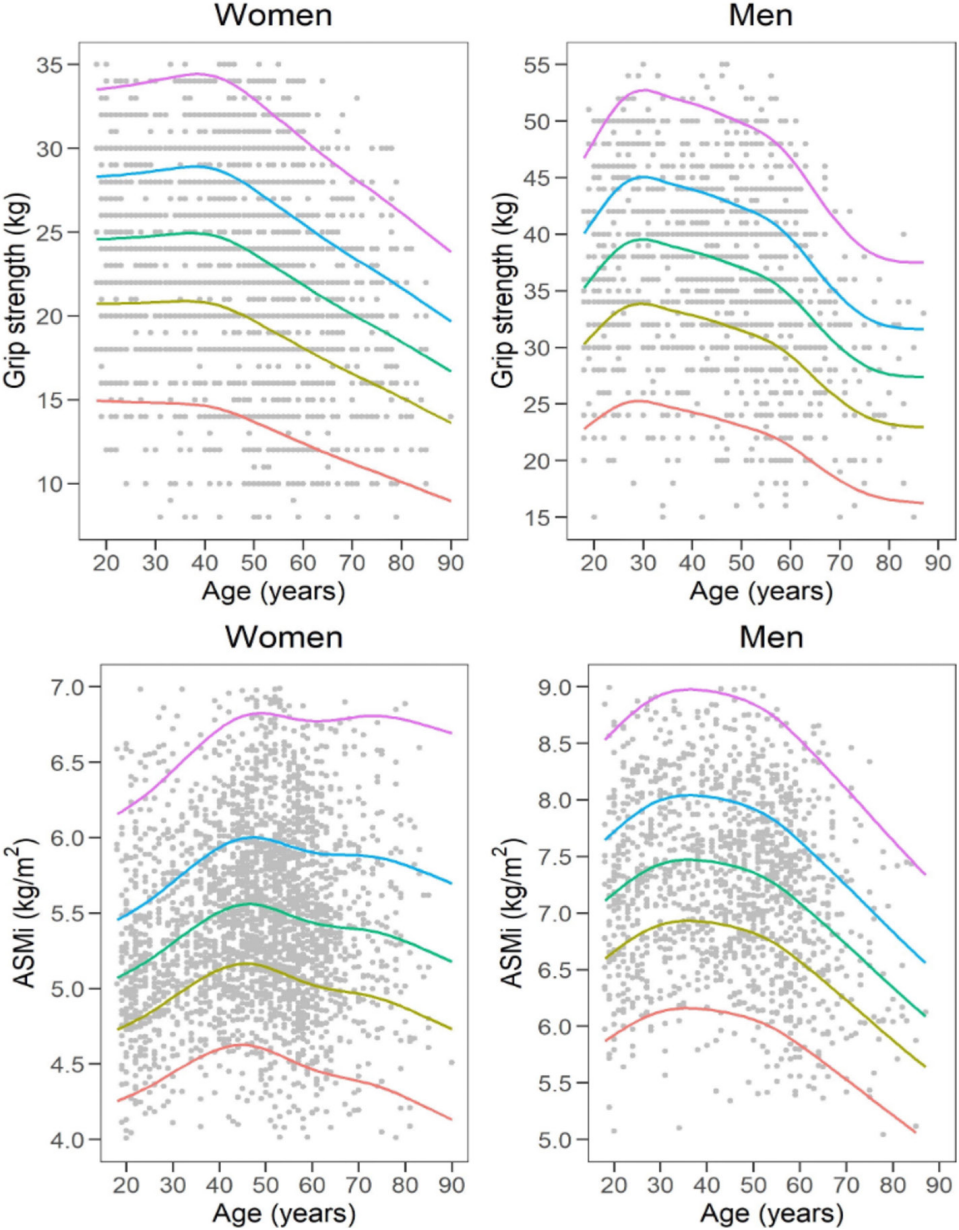
Handgrip strength and ASMI reference percentiles for women and men aged 18–80+ years. The 5th, 25th, 50th, 75th and 95th percentiles are shown in red, olive, green, blue and purple, respectively.

**FIGURE 2:**
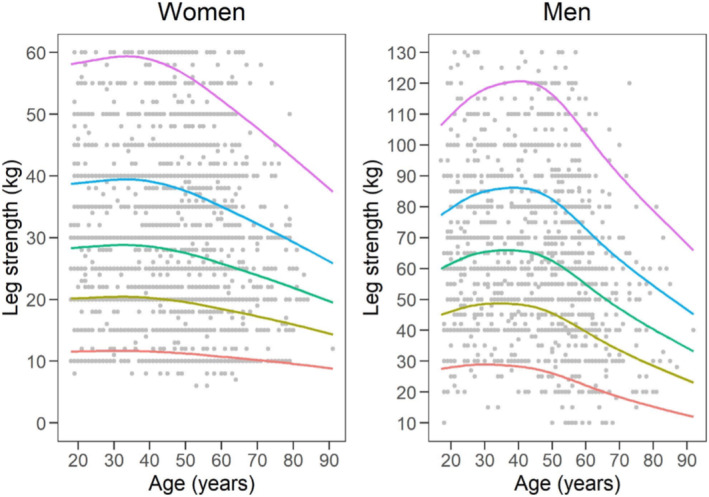
Leg strength reference percentiles for women and men aged 18–80+ years. The 5th, 25th, 50th, 75th and 95th percentiles are shown in red, olive, green, blue and purple, respectively.

To help translate these data in clinical practice, charts depicting the 5th, 25th, 50th, 75th and 95th percentiles across the age range from 18 to over 80 years have been constructed for both men and women. Additionally, charts illustrating the centile values for HGS in both genders are shown in Figures 1 and 2. Cut‐off values for sarcopenia diagnosis may be determined by the lowest quintile for muscle strength in the upper and lower extremities.

## 4 Discussion

Our study is the first to establish the age‐ and gender‐specific reference values for both handgrip and lower extremity strength across a broad age range using a large, representative sample of the Vietnamese population. We also developed ethnic‐specific thresholds for muscle strength and ASMI to aid in diagnosing sarcopenia in Vietnamese individuals. Our results indicate significant variations in the diagnosis of sarcopenia when our data are compared to the standards set by the AWGS. This highlights the critical need for ethnicity‐specific reference databases to accurately identify sarcopenia in various populations.

Various techniques are utilised for evaluating muscle strength, categorised into four main types: manual muscle testing (MMT), hand‐held dynamometry (HHD), isokinetic dynamometry and functional performance tests [22]. MMT has been traditionally used to assess strength but has limitations in detecting small changes, especially in patients with long‐term treatment or with athletes. Isokinetic dynamometry and functional performance tests provide reliable alternatives to MMT but are hindered by their large size, high cost, lack of portability and time‐consuming nature. In contrast, isometric muscle strength testing with HHD is a practical and effective method in a clinical setting due to its validity, ease of use and cost‐effectiveness [23].

HGS is commonly used to indicate overall muscle strength [24], but there is a controversy regarding its agreement with lower extremity muscle strength [25]. Additionally, lower limb performance appears to have a strong association with incident disability, particularly in older women [26] and in individuals with prevalent chronic diseases like osteoarthritis, diabetes and cardiovascular disease [27]. Tests assessing upper and lower extremity performance offer complementary insights into functional status. Previous studies have shown that the BLC dynamometer has high reproducibility and provides reliable measurements compared with functional performance tests [26], making it suitable for assessing lower limb muscle strength in this study.

Despite strong predictors of adverse negative health outcomes such as care dependence, falls, fractures, hospitalisation and death, muscle strength measures are not commonly assessed in daily practice compared to other clinical or biochemical parameters [24]. This limitation may stem from the need for more consensus on methods for evaluating muscle strength. However, based on numerous studies, HHD has been recommended for assessing muscle weakness in clinical and community settings because of its ease, speed, non‐invasiveness, affordability and reliability [28, 29, 30]. Additionally, normative reference values for HGS are essential for the practical interpretation. Although many normative reference values for grip strength have been published among Western populations [31, 32] and developed Asian countries [33, 34], there is still a need to establish normative values based on national specifics, considering ethnic and geographical variations [13].

Furthermore, recent meta‐analyses have highlighted the necessity for normative data in LMICs, as there is a lack of information available on the reference values in these countries [13, 14]. The first reference normative values for HGS and lower limb muscle strength among Vietnamese individuals address a critical research gap within LMICs and significantly contribute to our understanding of muscle strength reference data. Fortunately, the percentile charts produced by this study provide simple, rapid tools for assessment, which help interpret muscle strength evaluations in any care setting. This is particularly appropriate given the limited time available during a typical visit to a healthcare setting.

Although low muscle strength in sarcopenia has traditionally been linked with ageing and older individuals, it is now recognised that the onset of sarcopenia can occur earlier in life [35], and various factors beyond ageing influence its phenotype [31]. Notably, low muscle strength in adolescence and young adulthood has been strongly associated with an increased risk of premature death from various causes [36], as well as a heightened risk of cardiovascular disease [37] and Parkinson's disease [38] in later life. Addressing low muscle strength in youth could enhance adult muscular fitness and mitigate future chronic disease risks [39]. These insights emphasise the importance of early screening and intervention in adulthood to prevent or delay adverse outcomes later in life. For this practical purpose, our study's identification of a broad age range (from 18 to 90 years old) and gender‐specific normative values is valuable for detecting individuals with low muscle strength or those at risk for sarcopenia at a young age. This aligns with recommendations from the EWGSOP2 [11], and the EWGSOP2 algorithm can be effectively applied using the broad range of percentile charts provided in this study. As mentioned, if muscle strength values for specific age and gender categories fall below the 25th percentile, it could suggest abnormally low muscle strength, warranting further investigation.

We observed a relationship between muscle strength and advancing age that adhered to a third‐degree polynomial function, consistent with findings from previous studies [31, 32, 33, 34]. Muscle strength increased to a peak during early and middle adulthood, followed by a plateau phase, and subsequently declined with age. Although the age‐achieving *p*MS showed slight variation across studies, typically occurring during young and middle adulthood (20–40 years old) for both genders, there were notable differences in the actual *p*MS values. Compared to Caucasian and Asian populations, the *p*MS values for handgrip in our study, 39 kg in men and 25 kg in women, were lower than those reported in both populations. For instance, in British [31] and Italian [32] populations, the report values were 51 and 49 kg for men and 31 and 29 kg for women, respectively. Similarly, they were lower than the values reported in Korean [33] and close to Chinese [34] populations, which were 47 and 39 kg for men and 28 and 24 kg for women, respectively. Notably, for handgrip and lower extremity, the *p*MS values of the Vietnamese population (66 kg in men and 39 kg in women) were lower when compared to those of other countries [40, 41]. Although it is impossible to determine the underlying factors for this apparent difference, it is well known that the Vietnamese have different genetic backgrounds, anthropometric factors, dietary patterns, socio‐economic status and physical activity levels compared to other countries.

According to the recommendations of the AWGS, different cut‐offs are necessary for other ethnic groups [12], HGS is suggested for muscle strength measurement, and DXA is recommended for muscle mass evaluation [12]. Adapted to these suggestions, we have established parameters for sarcopenia diagnosis among the Vietnamese population. The lowest percentiles of HGS and ASMI for genders were utilised among the older population (> 60 years old), with the cut‐off values being 32 kg and 6.8 kg/m^2^ in men and 20 kg and 5.1 kg/m^2^ in women, respectively. Based on these derived cut‐offs, the prevalence of sarcopenia in Vietnamese men was 21% and in women was 14.2%, which differs from the 12.7% in men and 14.5% in women according to AWGS criteria. It is noteworthy that the existing sarcopenia definition by AWGS may underestimate the prevalence of sarcopenia in men of the Vietnamese population. These findings highlight the importance of ethnicity‐specific reference databases for accurately identifying sarcopenia.

The present results must be interpreted within potential strengths and limitations. One of the strengths is its contribution to addressing a significant public health concern in Vietnam, an Asian LMIC, by providing standardised reference values for muscle strength. The utilisation of a large sample size and broad age range representative of the Vietnamese population enhances the generalisability and reliability of the findings. Additionally, the construction of age‐ and gender‐specific percentile charts offers a user‐friendly approach that clinicians can utilise in their daily practice. We used standard methods HDD and DXA to assess muscle strength and muscle mass; we also evaluated lower extremity muscle strength by BLC dynamometer. However, it is essential to acknowledge some limitations of the study. The cross‐sectional nature of the data limits our ability to establish causal relationships or assess changes in muscle strength over time. Furthermore, the study primarily focused on handgrip and appendicular skeletal muscle strength, neglecting other important muscle groups contributing to overall physical function. Future prospective research should aim to include a more comprehensive assessment of muscle strength and explore additional factors influencing muscle health in the Vietnamese population.

## 5 Conclusion

Our study has provided valuable insights into muscle strength and sarcopenia among the Vietnamese population. By establishing specific reference values and cut‐off points for diagnosis, we have highlighted the importance of tailored approaches to address sarcopenia in diverse communities, particularly in LMICs. These normative values and percentile charts for muscle strength offer a standardised framework for assessing muscle strength and facilitate early detection and intervention, ultimately contributing to preventing and managing sarcopenia as a public health burden in Vietnam. Continuing research and clinical practice efforts are warranted to refine our understanding of muscle health and optimise strategies for preserving functional independence and quality of life among the Vietnamese population.

## Author Contributions

Kiet T. Do, Quan N. Luong, Duy K. Hoang, Lan T. Ho‐Pham and Tuan V. Nguyen designed and conducted the study. Kiet T. Do, Quan N. Luong and Huy G. Nguyen analysed the data. Kiet T. Do and Lan T. Ho‐Pham interpreted the data. Kiet T. Do, Quan N. Luong, Huy G. Nguyen, An T. Do, Duy K. Hoang and Lan T. Ho‐Pham prepared the manuscript and are responsible for the final content. All authors read and approved the final manuscript.

## Conflicts of Interest

The authors declare no conflicts of interest.

## Supporting information


**Data S1** Supporting Information.
