# Supplementary material for: Reference Values of Handgrip and Lower Extremity Strength for Vietnamese Men and Women: The Vietnam Osteoporosis Study
Source: J Cachexia Sarcopenia Muscle. 2025 Jan 10;16(1):e13689. doi: 10.1002/jcsm.13689 (PMC11718216; doi:10.1002/jcsm.13689)
Supplement: Supplementary file 1 — Data S1 Supporting Information. [file JCSM-16-e13689-s001.docx]

# Supplement

| Estimates of parameters of the third-degree polynomial regression model | | | | | |  |
| --- | --- | --- | --- | --- | --- | --- |
|  |  |  |  |  |  |  |
| **Muscle Strength** | **Parameters** | | | **R^2^** | **SEE** |  |
|  | **β₁(age)** | **β₂(age)²** | **β₃(age)³** |  | |  |
| **Dominant Hand** | | | | | |  |
| Men | -70.64 | -55.39 | 13.69 | 0.08 | 0.24 |  |
|  | (< 0.001 ***) | (< 0.001 ***) | (0.111) |  |  |  |
| Women | -62.09 | -37.87 | 7.24 | 0.06 | 0.12 |  |
|  | (< 0.001 ***) | (< 0.001 ***) | (0.221) |  |  |  |
| **Non - Dominant Hand** | | | | | |  |
| Men | -64.28 | -55.38 | 19.00 | 0.08 | 0.23 |  |
|  | (< 0.001 ***) | (< 0.001 ***) | (< 0.02 *) |  |  |  |
| Women | -51.48 | -41.74 | 7.84 | 0.05 | 0.11 |  |
|  | (< 0.001 ***) | (< 0.001 ***) | (0.171) |  |  |  |
| **Leg** | | | | | |  |
| Men | -157.79 | -139.32 | 30.05 | 0.04 | 0.79 |  |
|  | (< 0.001 ***) | (< 0.001 ***) | (0.288) |  |  |  |
| Women | -92.91 | -61.84 | 18.84 | 0.02 | 0.30 |  |
|  | (< 0.001 ***) | (< 0.001 ***) | (0.206) |  |  |  |
| 0 ‘***’ 0.001 ‘**’ 0.01 ‘*’ | | | | | |  |
| Values are coefficient (standard error) of the model MS = α + β₁(age) + β₂(age)² + β₃(age)³. | | | | | |  |
| R^2^, coefficient of determination indicates the proportion of variance in muscle strength of the dominant hand, non-dominant hand, and leg that could be "explained" by the polynomial model; SEE, Standard error of estimate. | | | | | |  |
